# Benign Mandibular Cavity/Stafne's Bone Cyst: A Case Report and Review

**DOI:** 10.1002/ccr3.71664

**Published:** 2025-12-09

**Authors:** Fawzia M. Butt, Shamim M. Butt, Mark L. Chindia

**Affiliations:** ^1^ Human Anatomy University of Nairobi Nairobi Kenya; ^2^ Human Physiology University of Nairobi Nairobi Kenya; ^3^ Oral and Maxillofacial Surgery University of Nairobi Nairobi Kenya

**Keywords:** aberrant salivary gland, benign mandibular cavity, idiopathic defect, Stafne's bone defect

## Abstract

Stafne's bone defect or benign mandibular cavity is a clinically asymptomatic, incidental radiolucent finding present in the body of the mandible. This report describes a high‐risk patient presenting with pain and a comparable radiological profile. This warranted surgical investigation to rule out the presence of an occult malignancy.

## Graphical abstract

A 3D reformatted mandibular left body image showing a well demarcated radiolucent lesion.

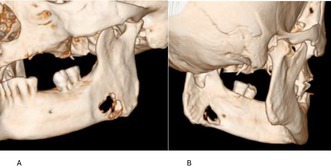

## 1 Introduction

Ectopic salivary gland tissue has been previously reported and described within various head and neck anatomical sites, including: the mastoid bone, middle ear, cervical lymph nodes, varied mandibular regions, and skin of the neck [1]. One unique case of ectopic salivary gland tissue is the Stafne bone defect (SBD) [2]. In 1942, Edward Stafne described this defect as being an anatomic rather than pathologic condition, whose probable cause is soft tissue inclusion. SBD is commonly found beneath the mandibular canal, between the first molar and angle of the mandible [3]. Diagnosed radiographically, it appears as circular, unilocular radiolucency, giving way to other preferred diagnostic terms, such as: Stafne's bone cyst/defect/cavity, mandibular salivary gland inclusion, ectopic/aberrant salivary gland, lingual mandibular bone cavity, static/idiopathic defects, or cavity [3, 4]. Aps et al. [5] proposed that SBD should be changed to “benign mandibular concavity” (BMC) since these asymptomatic lesions can present in any region of the mandible on a two‐dimensional radiograph [6]. In most cases, BMC comprises ectopic glandular tissue. However, muscle, lymphoid tissue, connective tissue, fat, soft tissue, or vasculature may also be found in the bone cavity [2].

BMC remain a rare finding; therefore, most often present as an asymptomatic incidental discovery during routine dental checks. BMCs have an incidence of 0.1%–0.48%, affecting mostly males in the fifth to seventh decades [5]. Stafne defects have been previously reported to be congenital anomalies. It is postulated that during mandibular development, the partial entrapment of the salivary gland tissue in the developing mandible causes such defects [5]. The authors state that when the submandibular gland is under pressure, this defect could arise as an auxiliary effect. It could also arise due to weakening or destruction via the adjoining lingual cortical plate [1].

The aim of the current report is to describe a patient complaining of a painful lesion that led to the suspicion of an occult malignancy. A unique presentation of a benign intra‐mandibular salivary gland inclusion manifestation.

## 2 Case History/Examination

A 66‐year‐old otherwise healthy male patient presented with localized, dull pain on the left side of the jaw for about 3 months. There was no reported use of pain medications, and his medical history was unremarkable. The patient reported smoking an average of 20 cigarettes daily and a glass of whiskey weekly for more than 50 years. Upon examination, extra and intraoral examination was normal, revealing no swelling on the left side of the mandibular region. On deep palpation, the area apical to the left lower molar region elicited an area of tenderness, although the overlying mucosa was normal.

## 3 Methods (Investigations, Differential Diagnosis and Treatment)

**FIGURE 1:**
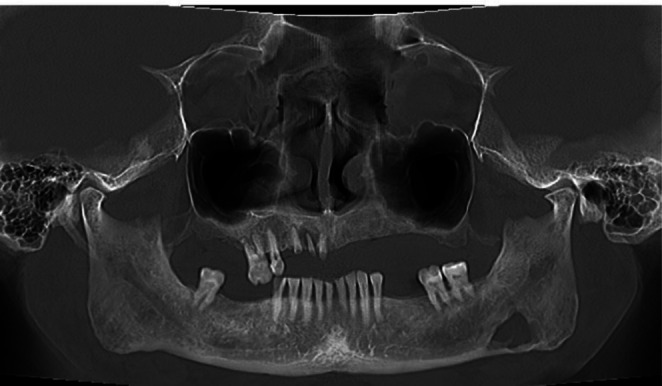
CT OPG A well‐demarcated radiolucent lesion in the left body of the mandible adjacent to the lower border.

**FIGURE 2:**
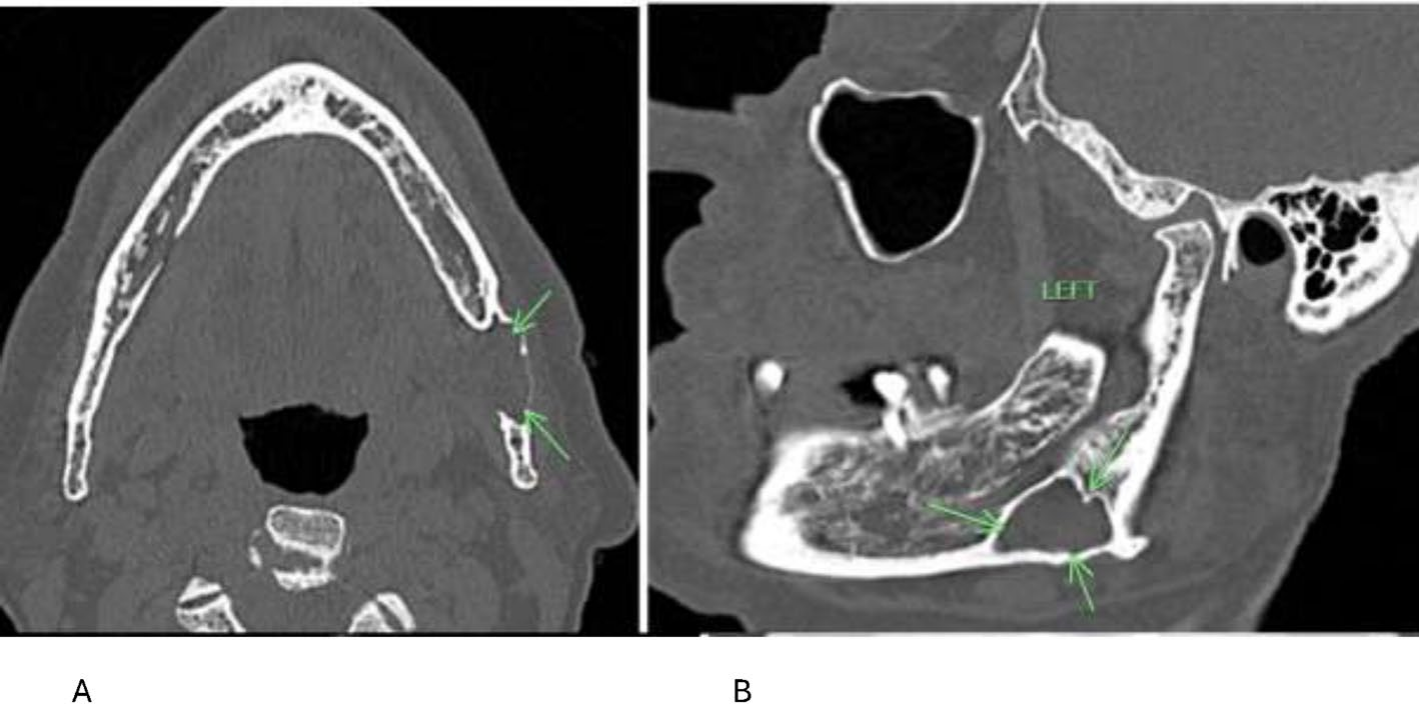
(A) Axial CT scan highlighting thinning of the buccal cortex. (B) Sagittal CT scan shows a sclerotic border outlining the radiolucent lucent lesion.

**FIGURE 3:**
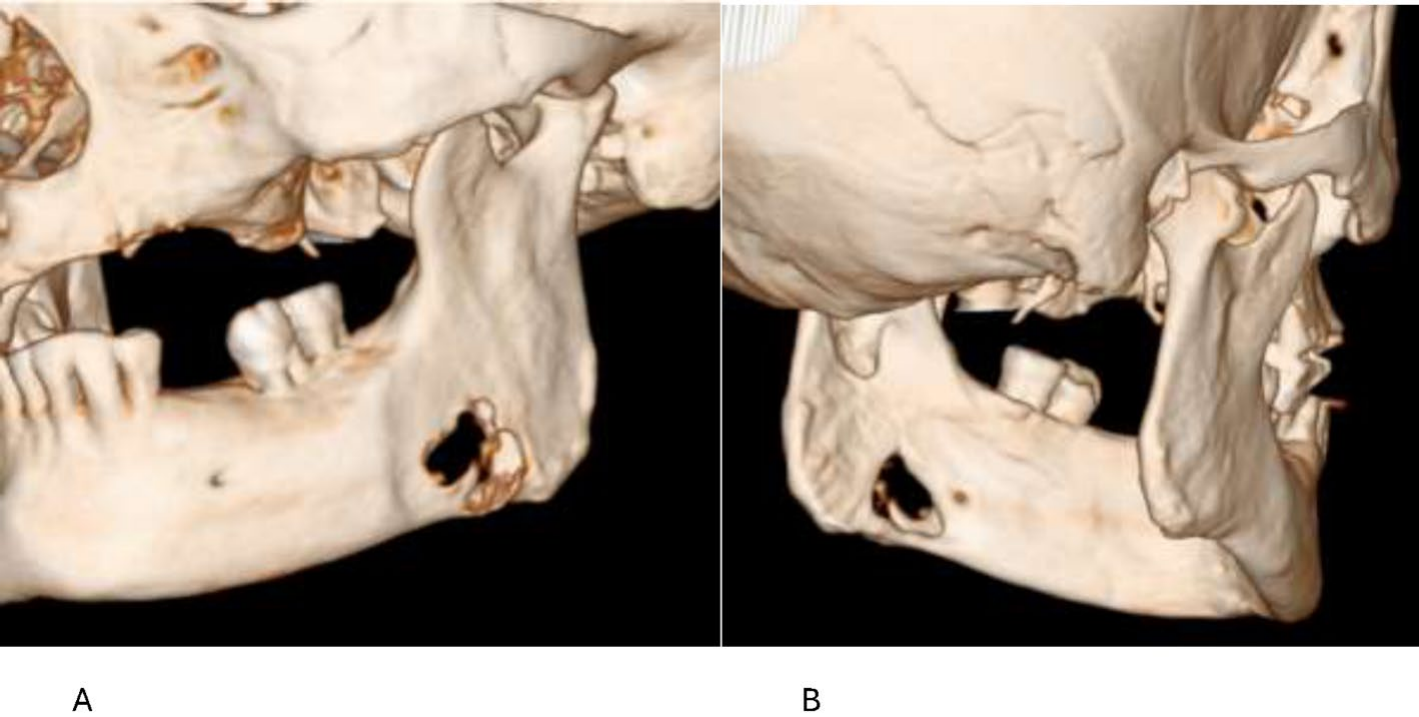
(A, B) 3D: Reformatted imaging illustrating the three‐dimensional erosion of the lesion in the body of the mandible both buccally and lingually.

The patient was sent for a Computed Tomography (CT) orthopantomogram (CT‐OPG) and CT scans—axial cuts and coronal cuts including 3D‐reformatted images (Figures 1 and 2A,B). The axial cuts highlighted thinning of the mandibular cortex, whereas the 3D‐reformatted images illustrated perforation of the buccal and lingual cortices inferior to the mandibular canal. The CT scans revealed a left‐defined unilocular corticated lytic non‐enhancing lesion that measured 22.6 mm × 9.0 mm × 13.4 mm {anterior posterior (AP) × transverse (TR) × craniocaudal l (CC)}, in the posterior aspect of the left mandible, adjacent to the angle of the mandible. The corticated lesion did not communicate with the mandibular canal and adjacent roots (Figure 3A,B). The differential diagnosis included occult metastatic malignancy, multiple myeloma, and MBC. Preoperative preparation included blood tests (full blood count, urea/electrolytes, and creatinine), high‐resolution chest X‐ray, CT of the abdomen, liver function tests, and electrocardiography, which were all normal. Under nasotracheal intubation, an incisional biopsy was obtained intra‐orally from the tender ramal region of the mandible.

## 4 Results

**FIGURE 4:**
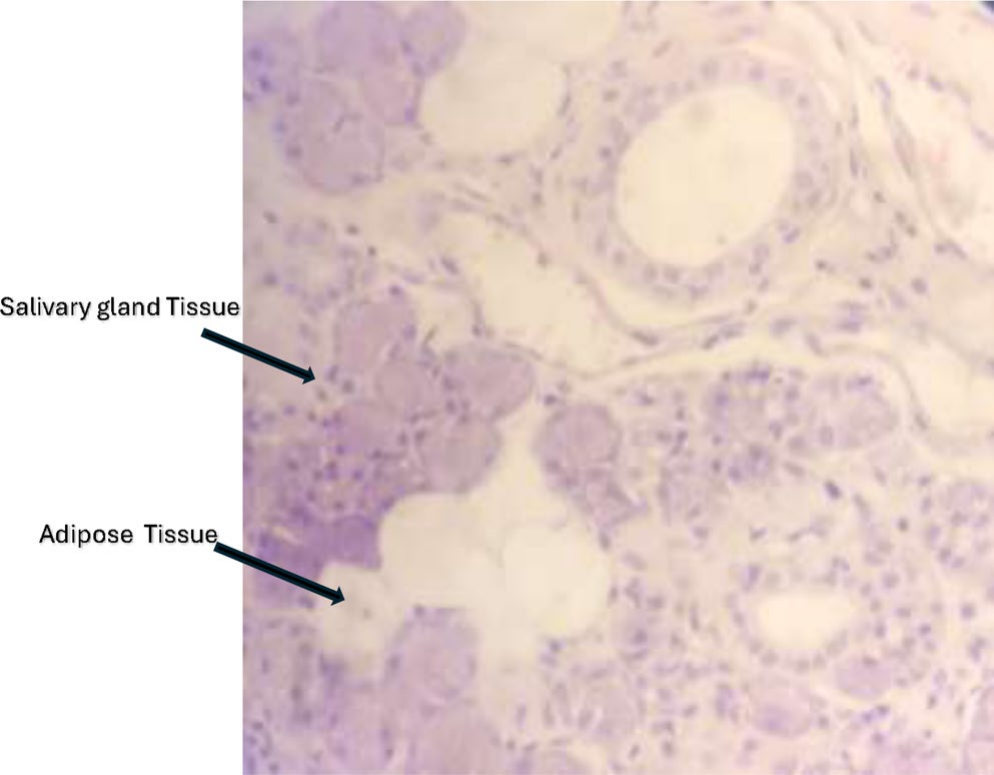
Hematoxylin and Eosin stain tissue (magnification ×40) showing salivary glandular tissue (presence of acini, ducts) and adipose tissue.

Histopathological examination reported it as a benign intra‐mandibular salivary gland inclusion. The macroscopic appearance was of multiple soft tissues 2 cm in diameter, and microscopically, the tissue consisted mainly of salivary gland tissue with benign ducts, acini, and adipose tissue. Additionally, no features of cell atypia were observed (Figure 4). The recovery was uneventful, and the patient's pain subsided postoperatively. The patient has been on review for the past 18 months with no complaints of pain, and the radiographic status of the lesion has been static.

## 5 Discussion

Clinicians abide by the Hippocratic oath (*primum non‐nocere*) [6]. Aggressive investigative approaches are ordinarily not adopted when the presentation is in keeping with all the radiological features of MBC. However, when the patient complained of dull pain, a biopsy was indicated due to his high‐risk behavior. Among the list of differentials, it was pertinent to rule out the presence of an occult malignancy due to his history of tobacco and alcohol consumption of more than 50 years. A normal CT scan of the abdomen, high‐resolution chest X‐ray, blood profile, and lipid function tests ruled out multiple myeloma and metastatic malignancy.

Radiological examination of the lesion gave it a classical description aligning with that of a BMC. The latter has been described as radiolucency dorsal to the mandible, inferior to the mandibular canal, above the cortical border of the mandible [7, 8, 9, 10, 11, 12]. Studies on the size of the bony defects have recorded values ranging from 0.5 to 2.0 cm with hardly any increases or decreases in dimension of the lesion, assuming more of a static nature [13, 14, 15]. In another study, volumetric measurements of BMC dimensions using Cone Beam CT showed ranges from 1 to 3 cm in diameter, depth (7.8 mm) and width (16.3 mm), respectively [16]. Although our patient had a CT scan, the lesion was reported to have a height of 13.4 mm and width of 22.6 mm, larger than the dimensions reported in the study by Adisen et al. [17]. There are two types of BMC, the anterior lingual variant (located amidst the incisors and the premolars, above the insertion of the mylohyoid muscle); the second is the posterior variant, which has been shown to be seven times more prevalent. The patient presented with the commoner posterior variant type [18, 19, 20]. BMC tends to present more in males than females (6:1–2:1) during the 4th to 6th decade of life with no racial bias [9, 21, 22, 23]. The age and gender of presentation in this patient was a 66‐year‐old male of Indian origin.

The etiology of BMC has been unclear with scanty histopathology evaluation of tissue. BMC has been a presumptive incidental radiological diagnosis due to its asymptomatic clinical presentation [5, 7]. The possibilities presumed that the erosion may have been caused by resorptive forces from a vessel, incomplete Meckle cartilage calcification during ossification, or an enlarged lobe of a salivary gland, or, as was suspected in this patient, by an occult malignancy initially. In our patient, the tissue was intramandibular, consisting of both salivary gland and adipose tissue, with no cell atypia reported, as “BMCs” are known to either contain salivary gland, lymphoid, muscular, adipose, and vascular tissue. Our histopathological report was similar, with two types of tissue found within the specimen: salivary and adipose tissue [7, 10, 12, 16, 21, 24, 25, 26, 27, 28, 29, 30, 31, 32, 33, 34, 35, 36, 37]. In general, these lesions are localized, non‐progressive, non‐healing, and are diagnosed as incidental findings with no clinical evidence. Therefore, often, a watch‐and‐wait approach is adopted with regular clinical and radio imaging [37].

The postoperative decrease in pain could be attributed to the release of nerve compression that may have been caused by the lesion. If the patient was in a low‐risk category with comparable incidental findings, a watch‐and‐wait approach with subsequent follow‐up would have been implemented.

## 6 Conclusion

In this patient with high‐risk behavior, surgical exploration followed by histopathology was pertinent due to the suspicious nature of the symptoms surrounding the BMC to rule out an occult malignancy.

## Author Contributions

**Fawzia M. Butt:** writing – original draft. **Shamim M. Butt:** writing – original draft, writing – review and editing. **Mark L. Chindia:** writing, review and editing.

## Funding

The authors have nothing to report.

## Consent

I confirm that I have obtained a written informed and signed consent form from the patient, and it has been uploaded as well.

## Conflicts of Interest

The authors declare no conflicts of interest.

## Data Availability

The data that support the findings of this study are available from the corresponding author upon reasonable request.
